# Heparan Sulfate Proteoglycan: An Arbovirus Attachment Factor Integral to Mosquito Salivary Gland Ducts

**DOI:** 10.3390/v6125182

**Published:** 2014-12-22

**Authors:** Kristen A. Ciano, Jason J. Saredy, Doria F. Bowers

**Affiliations:** Department of Biology, University of North Florida, Jacksonville, FL 32224, USA; E-Mails: kristenaciano@gmail.com (K.A.C.); sarj0007@ospreys.unf.edu (J.J.S.)

**Keywords:** *Alphavirus*, heparan sulfate proteoglycan, Aedine mosquitoes, salivary gland ducts, Sindbis virus, GFP

## Abstract

Variants of the prototype *Alphavirus*, Sindbis (SINV), were used in *per os* infections of adult female mosquitoes to investigate arbovirus interaction with the salivary gland (SG). Infection of Aedine mosquitoes with AR339, a heparan sulfate proteoglycan (HSPG)-dependent variant, resulted in gross pathology in the SG lateral lobes while infection with TR339, a HSPG-independent variant, resulted in minimal SG pathology. HSPG was detected in the internal ducts of the SG lateral lobes by immunolabeling but not in the median lobe, or beyond the triad structure and external ducts. Reports that human lactoferrin interacts with HSPG, suggested an interference with virus attachment to receptors on vertebrate cells. Pre-incubation of *Aedes albopictus* cultured C7-10 cells with bovine lactoferrin (bLF) followed by adsorption of SINV resulted in earlier and greater intensity of cytopathic response to TR339 compared with AR339. Following pre-treatment of C7-10 cells with bLF, plaques from tissue culture-adapted high-titer SINVTaV-GFP-TC were observed at 48 h post-infection (p.i.), while plaques from low-titer SINVTaV-GFP-TC were not observed until 120 h p.i. Confocal optics detected this reporter virus at 30 days p.i. in the SG proximal lateral lobe, a region of HSPG-immunolocalization. Altogether these data suggest an association between SINV and HSPG in the host mosquito.

## 1. Introduction

Arthropod-borne-viruses (arboviruses) are etiologic agents that contribute to significant diseases in humans and other animals. Sindbis virus (SINV), transmitted by hematophagous female mosquitoes, has caused outbreaks in humans every seventh year in Northern Europe [1] and can cause Pogosta disease, a systemic infection in humans resulting in arthritis, itching rash, fatigue, mild fever, headache, and muscle pain [2]. A member of the genus *Alphavirus* in the family *Togaviridae*, SINV is an enveloped virus with a plus-sense, single-stranded, 11.7 kb RNA genome [3]. In addition to being isolated from various insects and vertebrates in Northern Europe, SINV has been found in South and East Africa, Egypt, Israel, the Philippines, and parts of Australia [4]. Additionally, SINV has been isolated from Aedine, Mansonia and Anopheles mosquito species in nature [5,6] and demonstrated to replicate in *Aedes aegypti* and *Ae. albopictus* in the laboratory setting [7]. Replication of arboviruses in the mosquito host is essential for virus persistence, and a horizontal cycle is the primary mechanism of *Alphavirus* maintenance in nature [8]. Lack of evidence for a vertical transmission route for SINV indicates that feeding physiology of female mosquitoes is integral to arbovirus transmission.

Female mosquito salivary glands (SG) are paired organs located in the thorax, each consisting of three discrete lobes; two equivalent lateral lobes (LL) and one median lobe (ML). Each lobe has a central internal duct encircled by a monolayer of epithelial cells bounded externally by a basal lamina [9]. These three internal ducts fuse at a triad structure [10] that leads into two bilateral external main ducts (MD), which coalesce to form an external common salivary duct that opens at the base of the hypopharynx [11]. Salivary glands are essential to mosquito blood feeding behavior, and paramount to virus propagation by bite. Investigation into the SG biochemical and structural differences [12,13,14] as well as virus-associated SG pathology has provided insight into arbovirus transmission in nature. Bowers and colleagues [15,16] demonstrated that SINV replicated to high titer in the whole insect and presented structural of SINV-associated pathology in the SG following intrathoracic inoculation in *Aedes albopictus*. Colocalization of gross organ disruption, virus antigens, and apoptotic cells [10] were detected in the proximal and distal LL, while neither structural nor immunological evidence of SINV were observed in ML.

Because *Alphaviruses* have a broad host range in nature, replicating in mammalian, avian, arthropod and amphibian species [17], it has been suggested that a universally-expressed molecule is essential for attachment. Adaptation of SINV to growth in tissue culture or in animals has generated mutants that can be used to evaluate strain-specific differences in receptor usage [18]. Following passage in the mammalian cell line, BHK-21, SINV has a positively-charged amino acid substitution in the virus spike protein E2, which permits attachment to HSPG [19]. Localized on the cell surface of most eukaryotic cells, HSPG has a net negative charge that plays a significant role in *Alphavirus* attachment [18] and has been detected in mosquito SG by disaccharide analysis [20]. Since *Alphavirus* virions bind to receptors that are highly conserved between species, it was suggested that binding of human lactoferrin (hLF) to cell-surface HSPGs may inhibit virus infection [21]. Human LF is an 80-kDa cationic glycoprotein produced by epithelial cells and found in mucosal secretions such as tears, saliva, gastrointestinal fluids, and human breast milk [22]. Preabsorption of cultured vertebrate cells with hLF strongly inhibited infection of cells by HS-adapted *Alphaviruses*, such as Semliki Forest virus (SFV), but not the non-adapted SINV strain TR339 [19]. The acute phase of SINV infection in *Ae. albopictus* larval cell line C7-10 resulted in a cytopathic effect (CPE) that led to high death rates similar to that observed in BHK-21 cells [23] and the ability of LF to decrease infectivity of SINV in C7-10 cells was evaluated. This current investigation used variants of SINV in an attempt to correlate the presence of biochemical differences within the mosquito salivary gland lobes by localization of HSPG and SINV to precise regions.

## 2. Results

### 2.1. Virus-Associated Pathology in Salivary Glands

**Figure 1:**
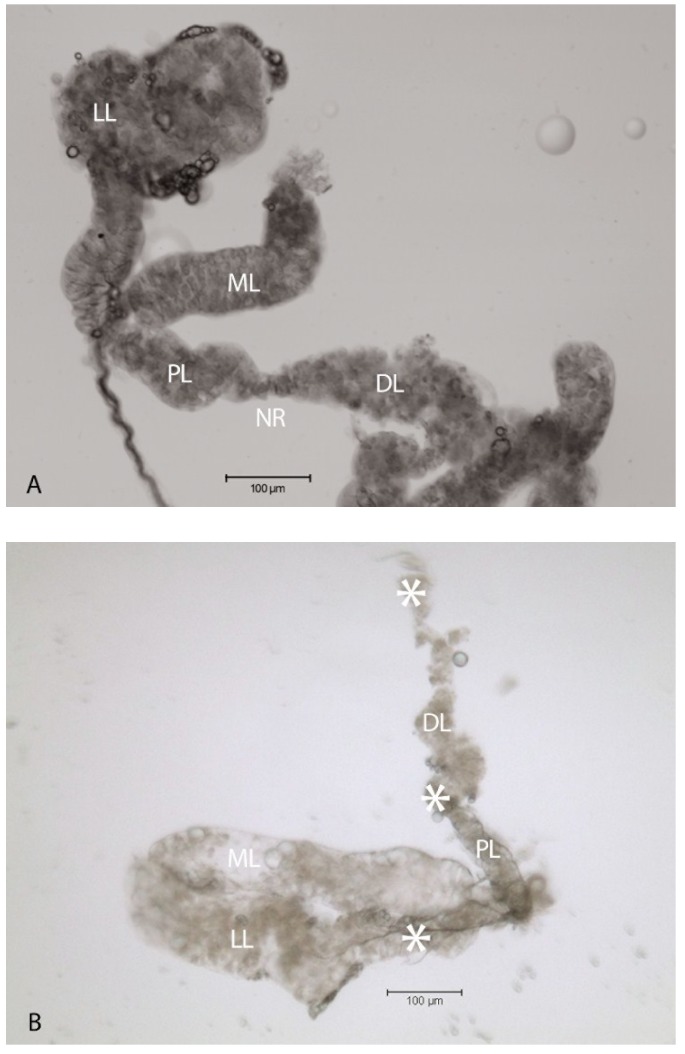

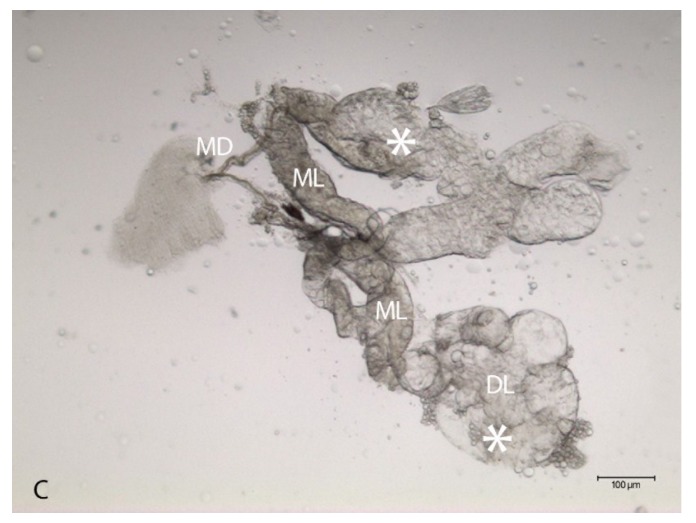
SINV-associated pathology in SG’s dissected from adult female *Ae. aegypti* mosquitoes. (**A**) Bright field image of a SG from an uninfected mosquito. The lateral lobes (LL), proximal lateral lobe (PL), distal lateral lobe (DL), neck region (NR) separating PL from DL and median lobe (ML) are labeled for orientation purposes; (**B**) Bright field image of a SG dissected at 14 days p.i. with AR339 *per os*. The DL and PL appeared grossly cytopathic (*), one LL was completely disrupted and absent, while the ML remained intact; (**C**) Bright field image of a SG dissected at 28 days p.i. with TR339 *per os*. Two sets of SG’s are displayed, connected by the two main ducts (MD), some CPE (*) is observed in the DL, while the two ML appear intact.

Cytopathology in the LL of the SG following intrathoracic inoculation with SINV has been documented in *Ae. albopictus* [16] and these results were reproduced in *per os* infections of *Ae. aegypti* in this study. Resected SG’s from AR339-blood fed individuals showed distention and disruption of the LL at day 14 post-infection (p.i.) (Figure 1B) compared with SG’s from uninfected mosquitoes (Figure 1A). Comparatively, LL specific pathology was minimal in TR339 *per os* infected mosquitoes (Figure 1C) at 28 days p.i. The ML remained intact without pathology, in response to virus variants and all mosquitoes used for Figure 1B&C had positive CPE leg-assays indicative of virus dissemination. 

### 2.2. Localization of HSPG to Salivary Glands

**Figure 2:**
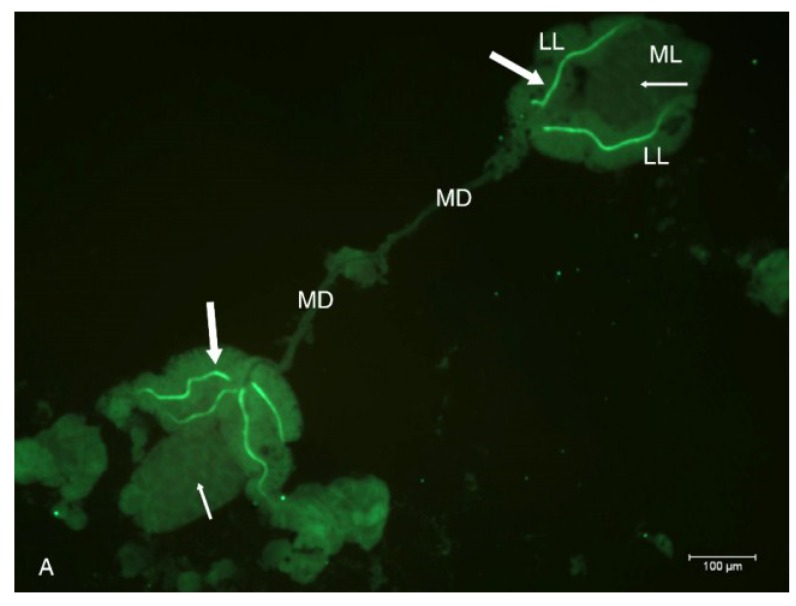

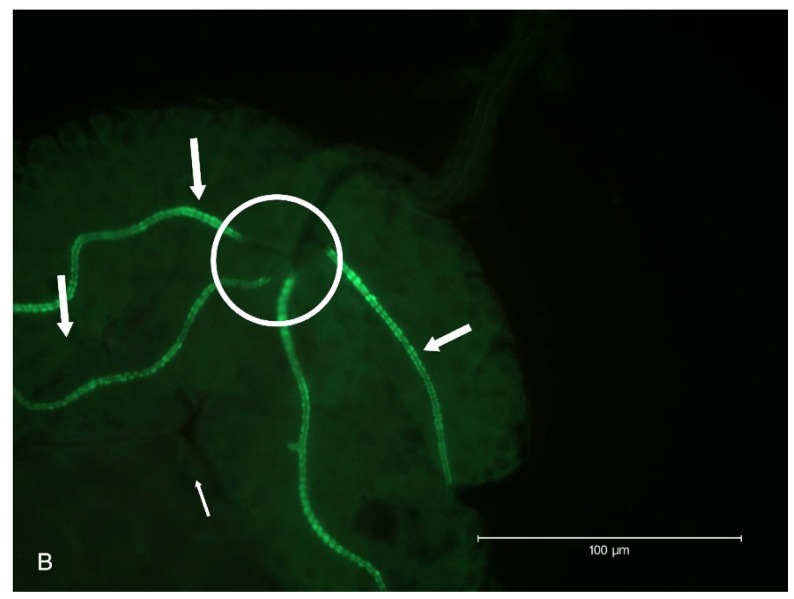
Epifluorescent immunodetection of HSPG to SG ducts from *Ae. albopictus*. (**A**) Fluorescence image of two sets of SG’s immunolabeled for HSPG using monoclonal antibody C17. Two main ducts (MD) are apparent. Each single tri-lobed SG is composed of two longer lateral lobes (LL) and a single shorter median lobe (ML). Internal ducts (thick arrows) stained positive for HSPG in the LL, and internal ducts (thin arrows) in ML are deficient of detectable HSPG; (**B**) Higher magnification of a SG labeled for HSPG. This aberrant SG has four LL’s instead of the usual two; hence the triad structure (circle) has five instead of three internal ducts entering the MD ([10]; See SG diagram). Positive fluorescence signal localized HSPG to filamentous extensions of duct cuticle in LL’s appeared as “beads-on-a-string”.

Immunohistochemistry labeling of HSPG on whole mount preparations of *Ae. albopictus* SG detected HSPG in the SG ducts of the LLs (Figure 2A). The HSPG antibody appeared to have localized to the duct epithelial cell filamentous extensions lining the duct lumen in the LL (Figure 2B), while the median duct was HSPG-deficient.

**Figure 3:**
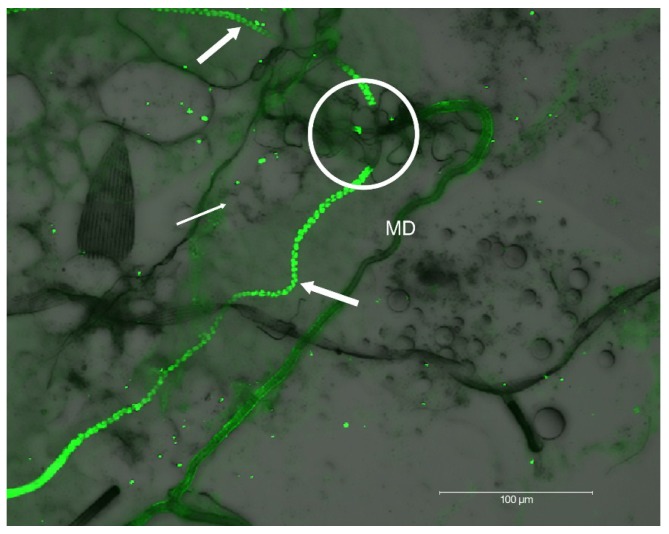
DIC microscopic analysis of HSPG immunolocalization to internal ducts of SG’s dissected from *Ae. albopictus*. Note distinct labeling of HSPG to LL’s internal ducts (thick arrows), absence of detection in ML (thin arrow), the abrupt absence of HSPG at the triad structure (circle), and the lack of detectable HSPG in the external SG main ducts (MD).

Immunofluorescence differential interference contrast (DIC) microscopic analysis of SG revealed an abrupt absence of HSPG at the triad structure where internal ducts fuse to form a single main duct (Figure 3). This immunolabeling pattern observed at the triad, accentuates the internal and external duct junction.

### 2.3. Lactoferrin Inhibition of SINV Infection in C7-10 Cells

**Figure 4:**
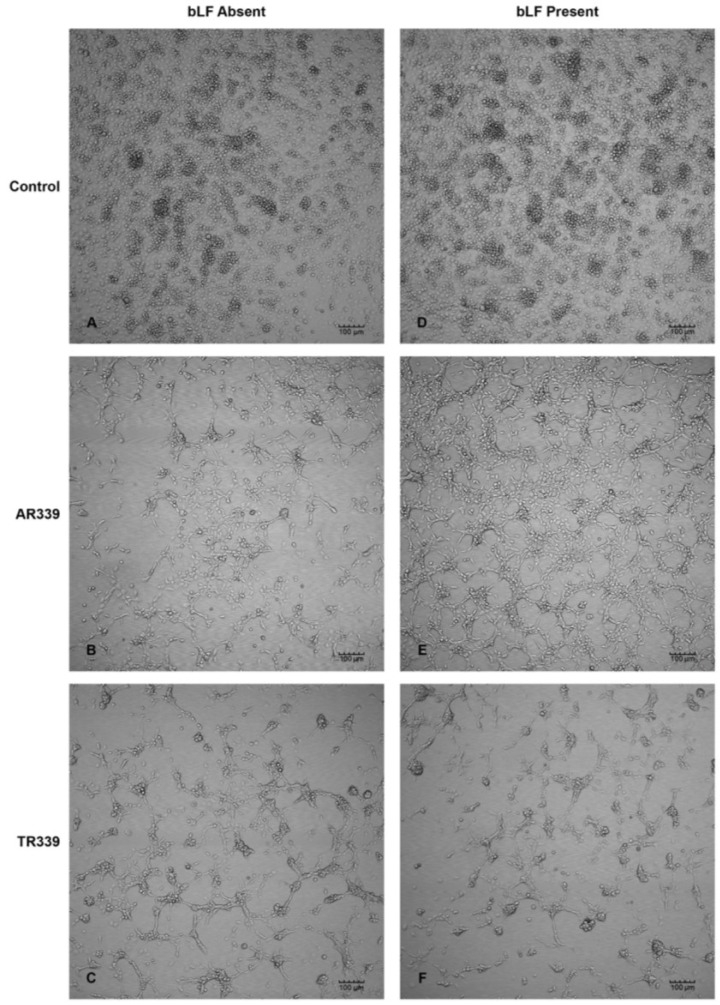
Inhibition of SINV-associated CPE in C7-10 mosquito cells at 48 h p.i. in response to bLF. (**A**,**D**) Controls, no virus added. Cells remain healthy in the absence of bLF (**A**) and after addition of 200 µg/mL of bLF (**D**); (**B**,**E**) SINV challenge using AR339 (M.O.I. of 0.1). CPE is observed in cells infected with AR339 in the absence of bLF (**B**), but CPE is less extensive following addition with 200 µg/mL of bLF (**E**); (**C**,**F**) SINV challenge using TR339 (M.O.I. of 0.1). Equivalent CPE is observed in the absence of bLF (**C**) and in the presence of 200 µg/mL of bLF (**F**).

Preincubation of C7-10 cells with bLF (200 µg/mL) or cell culture media (absence of bLF, negative control) was followed by adsorption of virus at a multiplicity of infection (M.O.I.) of 0.1 (Figure 4). At 48 h p.i. CPE was not observed in both controls (mock infected), CPE appeared equivalent in response to TR339 (HSPG-independent) infection, while CPE was less extensive following bLF pretreatment and infection with AR339 (HSPG-dependent) compared with the absence of bLF control. Cells pretreated with bLF followed by infection with AR339 (E), appeared more attached to the substratum and less flask surface was visible when compared with cells not pretreated with bLF (B).

### 2.4 Confocal Analysis of Plaque Assays and Salivary Glands

The reporter virus construct, SINVTaV-GFP, was created by inserting the GFP genetic sequence into TR339 [24]. Tissue culture adapted SINVTaV-GFP-TC was created by multi-passage in BHK-21 cells (see Section 4.2). This TC-adapted virus was used in plaque assays on mosquito cells; C7-10 cells were pre-incubated with 200 µg/mL bLF in Eagle’s minimal essential medium supplemented with 10% fetal bovine serum (FBS; Gibco, Carlsbad, CA, USA), 10% tryptose phosphate broth, 20 µg/mL Gentamycin (EMEM), or in EMEM alone prior to exposure to SINVTaV-GFP-TC at a M.O.I. of 0.1 in the absence or presence of bLF (Table 1). In the absence of bLF, a very small focus was visible at 24 h p.i., three medium plaques following 48 h p.i., and three large plaques at 120 h p.i. In the presence of bLF small plaques were not observed until 120 h p.i. indicating an inhibitory effect of bLF on this TC-adapted variant on cultured mosquito cells. These results suggest that multiple passage of TR339 changed from HSPG-independent to HSPG-dependent phenotype.

**Table 1:** Lactoferrin inhibition of SINVTaV-GFP-TC plaque-formation at M.O.I. of 0.1.

	24 h p.i.	48 h p.i.	120 h p.i.
No bLF	1 small foci *	3 medium plaques	3 large plaques
200 µg/mL bLF	No plaques	No plaques	4 small plaques

Multiplicity of infection (M.O.I.), post-infection (p.i.), bovine lactoferrin (bLF); * Average number of plaques per flask (*n* = 3) on C-710 cells.

**Figure 5:**
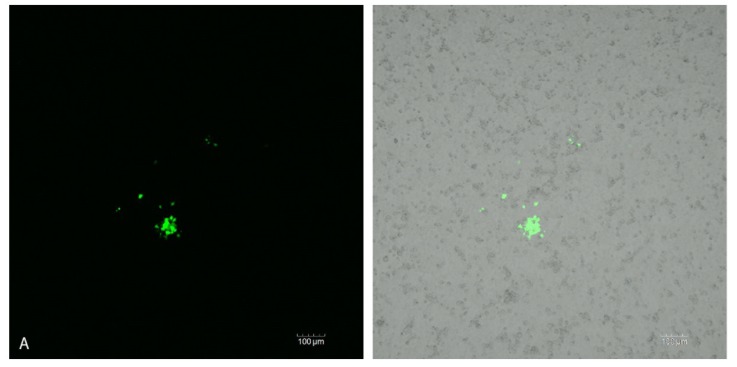

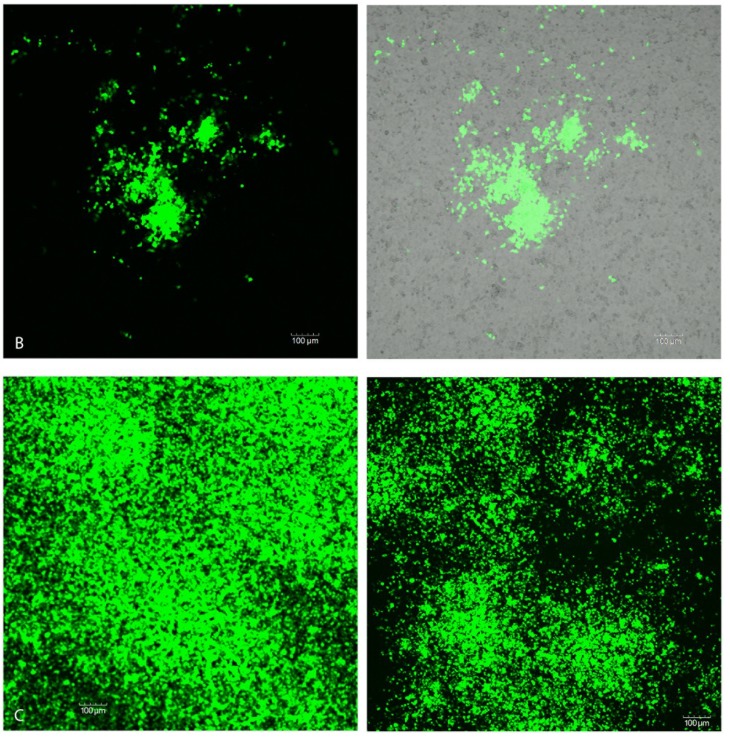
Immunofluorescence confocal microscopy of SINVTaV-GFP-TC infection of C7-10 cells monitored over time in the same culture plates. Composite images; immunofluorescent (left) compared with immunofluorescent merged with phase contrast (right) revealing background monolayer (**A**,**B**); Plaque growth in cells infected with a M.O.I. of 0.1 in the absence of bLF at (**A**) 24 h p.i. and (**B**) 48 h p.i.; (**C**) Plaque growth in cells infected with a M.O.I. of 100 at 48 h p.i. in the absence (left-image) *vs.* presence bLF (right-image).

This inhibitory effect of bLF against SINV in cultured mosquito cells was further investigated by confocal microscopy. Plaque size at different times post-exposure in the absence of bLF (Figure 5A,B) and bLF effects of high titer plaque formation at 48 h p.i. (Figure 5C) was investigated. High M.O.I. challenge using TC-adapted TR339 on bLF pretreated C7-10 cells resulted in delayed virus growth.

**Figure 6:**
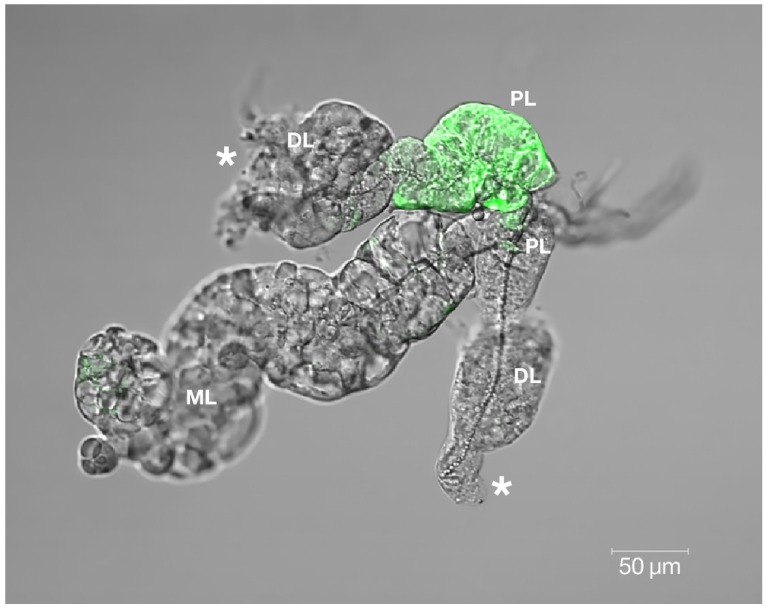
Confocal immunomicroscopy revealed SINVTaV-GFP-TC persistence localized in the SG proximal lateral lobe (PL) at day 30 p.i. on this whole-mounted specimen. Gross cytopathology (*) was observed in both distal lateral lobes (DL). Virus GFP was not detected in the median lobe (ML), which appeared intact and comparable to the uninfected control ML (Figure 1A).

Cytopathology and persistence of GFP reporter in the SG proximal lateral (PL) lobe was observed at day 30 p.i. following *per os* infection of *Ae. aegypti* with SINVTaV-GFP-TC variant (Figure 6). Both of these persistent virus-associated changes are located in the LL while the ML appears intact. Observation of GFP in the PL suggests retention of virus, retention of the GFP protein or both.

## 3. Discussion

*Per os* infection of *Ae. aegypti* with SINV resulted in SG LL-specific pathology that was more pronounced following AR339 compared with TR339. Oral infection with virus variants more accurately portrays virus-host interactions “in nature” compared with intrathoracic inoculation and suggests that the degree of CPE may differ between variants. Lateral lobe disruption, juxtaposed to intact ML suggests important biochemical and structural intra-organ differences. Another *Alphavirus*, SFV, caused gross morphologic changes in the salivary glands of *Ae. aegypti* [25]. Regional discrepancy in virus-associated pathology within the mosquito SG may provide clues to the transmission of these disease agents between species. Published studies [16,26,27,28] suggested that the ML may be essential for insect survival and reproduction, speculating that significant pathology of the LL might not be as lethal to insect survival and fitness as previously thought.

The SINV consensus sequence, TR339, attaches initially to cells through a low affinity, primarily HSPG-independent mechanism [18]. In contrast, AR339 utilizes cell-surface HSPG molecules as attachment receptors in a HSPG-dependent manner [18,29]. Interaction of viruses with HSPG, a result of tissue-culture adaptation, has been reported for several viruses [30,31,32], including clinical isolates of other viruses (echoviruses, herpes simplex virus) as well [33,34]. While virus-binding to cell surface HSPG is not equivalent to interactions between natural SINV isolates and cells *in vivo*, it does offer a means to study the mechanisms of other HSPG adapted arboviruses, such as dengue viruses and eastern equine encephalitis virus. Our initial hypothesis speculated that HSPG would be localized at the basement membrane surrounding the acinar epithelial cells of the SG LL’s. Instead this molecule was localized to the LL internal ducts, specifically studded on filamentous extensions of the duct cuticle (Figure 2A,B and Figure 3). Ultrastructural analysis of the internal ducts of the LL were described as containing a lumen of about 1.2 µm in diameter, duct wall about 0.5 µm thick, and duct thickenings observed by light microscopy were described as regions of fine filamentous extensions of the duct cuticle [13,14]. Maze-like canals (0.1 µm in diameter) penetrate the duct in the regions of the filaments and we believe that it is these filamentous short membrane extensions [35,36] that labeled positive for HSPG (Figure 2A,B and Figure 3). The ML duct structure differs from the LL ducts by its lack of these duct cuticle filamentous projections and the absence of HSPG immunolabeling confirmed this biochemistry. Alternately, the characteristic “beads-on-a-string like” HSPG labeling (Figure 2B and Figure 3) may be associated with the basement membrane of the LL duct epithelium. Because of the abrupt lack of HSPG localization at the triad structure (Figure 2 and Figure 3); we can likewise surmise a difference in the MD. Quite possibly the LL internal ducts are specialized for secretion while the internal ducts of the ML and external ducts of the MD are specialized for saliva transport. Future research at the ultrastructural-level would be vital to resolve fine-labeling of sub-duct structure.

While these data do not offer an initial route of SINV entry into the SG, these experiments do provide a clearer picture of biochemical differences between the LL and ML of the SG in adult female Aedine mosquitoes. Prominent LL pathology would appear to be a disadvantage to the female mosquito, affecting her ability to produce saliva, and therefore feed and reproduce. The acinar cells destroyed by the virus, however, may not constitute the sole source of saliva in the mosquito. Muangman [37] documented the transmission of SINV to suckling mice following transection of SG ducts, suggesting the passage of secretions across the duct wall. Their findings that “duct-transected mosquitoes” imbibe blood caused these researchers to conclude that saliva is not a prerequisite for blood feeding and virus transmission. Rossignol and Spielman [36] hypothesized that the transmitted virus may have not been aspirated from the hemolymph near the open ends of the transected ducts, but instead originated in the duct wall. This would coincide with the results presented in the current study, illustrating obliteration of the gland acinar cells, while the internal duct remains intact (Figure 1B and Figure 6).

Studies aimed at inhibiting virus interaction with HSPG utilizing the 80-kD cationic glycoprotein LF are becoming more prevalent [21,38,39]. Pre-treatment of C7-10 Aedine mosquito cells with bLF resulted in reduced CPE when infected with AR339, the HSPG-dependent variant (Figure 4B,E), compared with TR339, the HSPG-independent variant. Cells exposed to TR339 presented equivalent characteristics of CPE at 48 h p.i. without or with bLF (Figure 4C,F). When utilizing a TC-adapted TR339 variant (SINVTaV-GFP-TC) in mosquito cell plaque assays, virus propagation was temporally limited in the presence of bLF (Table 1; Figure 5). Similarly, Waarts and colleagues [19] demonstrated that hLF strongly inhibited infection of vertebrate cells (BHK-21) by HSPG-adapted *Alphaviruses* (70% reduction in virus plaques for TRSB, and 90% reduction for SFV)*,* but no reduction for non-adapted TR339. Together, this suggests that bLF inhibits TC-adapted SINV infection because these variants use HSPG for attachment and the reporter variant, SINVTaV-GFP-TC, is HSPG-adapted because of its passage history (five passages in BHK-21 cells). The method of tagging TR339 via the TaV-like catalase results in a TR-339-TaV-GFP that is effectively wild-type TR339 with the additional GFP-tag in the genome [24]. Capsid and E1/E2 are identical to wild type TR339, hence the need for the multi-passages in assure both TC-adaptation and retention of GFP. Localization of fluorescence in the SG PL at day 30 p.i. following oral inoculation demonstrated persistence of GFP protein originating from a TC-adapted variant in a HSPG-containing region of the SG. Prominent CPE in the LL ensures virus access to the SG by creating an “open-door” (Figure 1B and Figure 6) and HSPG may act as a “sponge” retaining virus for persistent transmission. Sindbis virus has long been an important tool to investigate arbovirology and the results of this study offer insight into interactions between an arbovirus and its mosquito host at the tissue-organ level.

## 4. Materials and Methods

### 4.1. Cell Culture

BHK-21 cells were grown in EMEM (ATCC, Manassas, VA, USA) supplemented with 10% fetal bovine serum (FBS; Gibco, Carlsbad, CA, USA), 10% tryptose phosphate broth, 20 µg/mL Gentamycin (Fisher Scientific, Suwanee, GA, USA) and maintained in culture at 37 °C with 5% CO_2_ [40]. *Aedes albopictus* cultured C7-10 cells (provided by Dennis T. Brown, North Carolina State University, Raleigh, NC, USA) were grown in EMEM supplemented as above with addition of 2.5 µg/mL Amphotericin-B (Sigma Scientific, Suwanee, GA, USA) and maintained in culture at 28 °C with 5% CO_2_ [40,41].

### 4.2. Virus Production

AR339 was obtained from ATCC (Manassas, VA, USA), TR339 (consensus sequence kindly provided by Brown/Hernandez Research Group, North Carolina State University, Raleigh, NC, USA) was generated from a cDNA clone and SINVTaV-GFP (reporter virus kindly provided by William Klimstra, University of Pittsburgh, Pittsburgh, PA, USA) were provided as viable constructs. The reporter virus protein GFP gene sequence was inserted as a fusion capsid reporter into wild type SINV consensus strain, TR339 [24]. The first five amino acids of E3 were inserted in-frame to the reporter and the reporter was followed by *Thosea asigna* virus (TaV) 2A-like protease. This creates an in-frame reporter protein with a leading capsid auto-protease and trailing TaV 2A-like protease that permits the cleavage of the reporter protein in the cytoplasm of infected cells. These progeny viruses are identical to wild type TR339. In order to achieve tissue culture adapted virus, cultured BHK-21 cells were grown to pre-confluence in 25 cm^2^ flasks. A 200 μL dilution of expression reporter virus was adsorbed on BHK-21 cells for 1 h, washed with PBS-D and replaced with 3 mL of EMEM. Virus was grown for 24 h, media was harvested, spun-down at 2000 rpm for 10 min and supernatant contained passaged virus that was titered via plaque assay in parallel with each increased passage number. Tissue culture adapted SINVTaV-GFP (SINVTaV-GFP-TC), was generated through 5 passages in BHK-21 cells to a final titer of 1.5 × 10^8^ pfu/mL. All experimentation was conducted in biological safety level 2 laboratories.

### 4.3. Growth and Maintenance of Colony Mosquitoes

Colonized *Ae. albopictus* (Lake Charles Strain) were maintained at 25.5 °C ± 0.5, 70%–80% relative humidity and a 16:8 (light/dark) photoperiod in white plastic bucket cages with mesh lids with no more than 350 mosquitoes per cage [42]. Mosquito eggs (United States Department of Agriculture, Gainesville, FL, USA) were hatched in 1.0% nutrient broth (1 gm/100 mL tap water and first instar larvae were distributed approximately 300/pan of 1.5 L tap water and fed a 2% aqueous liver powder suspension (Fisher Scientific, Suwanee, GA, USA). Adults were supplied with honey-soaked cellucotton as a carbohydrate source and water-soaked cotton *ad libitum* [43].

### 4.4. Artificial Membrane Feeding and Virus Infection of Mosquitoes

For colony maintenance, mosquitoes were proffered defibrinated bovine blood (Colorado Serum, INC, Denver, CO, USA) at 5 to 7 days post-emergence. For virus infections, free-mated adult female mosquitoes were provided a viremic blood suspension via an artificial membrane system [44]. At 72 h prior to blood feeding, cohorts of 50 females were placed in each cage and carbohydrate meals were removed at 24 h before feedings. AR339 and TR339 were diluted in EMEM to provide a final titer of 7.4 × 10^7^ pfu/mL in blood meals. After feeding on viremic or non-viremic (control) blood for 1 h, fully engorged mosquitoes were gently vacuum-aspirated into new cages and maintained as described above.

### 4.5. Leg Assay for Determination of Virus Dissemination

Legs from viremic-fed and non-virus-fed (negative control) mosquitoes were removed following prescribed days p.i. [45]. Individual legs from individual mosquitoes were placed in 6-well plates of BHK-21 cells grown in EMEM with the addition of Amphotericin-B (2.5 μg/mL). Culture plates were incubated (See Section 4.1 above), and observed for CPE. Presence of CPE was indicative of a disseminated virus infection and absence of CPE indicated lack of dissemination.

### 4.6. Salivary Gland Isolation and Localization of HSPG

Non-blood fed female mosquitoes were cold anesthetized, placed on a glass microscope slide with a few drops of cold phosphate buffered saline (PBS) while viewed through a Leica dissection stereomicroscope (Bannockburn, IL, USA). Salivary glands were removed, and whole-mount preparations were fixed in 4% paraformaldehyde/0.1 M Na cacodylate buffer (pH 7.3) (Fisher Scientific) for 1 h at room temperature (RT) followed by 3 PBS rinses. Tissues were stored at −20 °C prior to immunohistochemistry.

All immunohistochemistry labeling assays were performed in a humidity chamber; whole-mount SG were placed on glass slides and incubated in blocking solution (10% Normal Goat Serum (NGS); Gibco, Grand Island, NY, USA, in PBS) for 30 min at RT. Next, tissues were incubated in 250 µL of the primary antibody, HSPG C17 (mouse anti-rat HSPG basement membrane, IgG monoclonal antibody; Hybridoma Bank, Iowa City, IA, USA), at a 1:100 dilution in PBS containing 2% NGS, for 2 h at RT. All tissues were washed with PBS and incubated with 250 µL of secondary antibody, FITC-conjugated goat anti-mouse IgG (Sigma-Aldrich, St. Louis, MO, USA), at a 1:60 dilution in PBS containing 2% NGS, for 30 min at RT. Again, tissues were washed and slides were mounted and cover slipped with 0.25 mL VECTASHIELD (Vector Labs, Burlingame, CA, USA), and observed using an Olympus BX60 Epifluorescence microscope (Tokyo, Japan) equipped with a U-MWU dual filter. A KE/SE digital camera captured the images, which were collected using a SPOT/RT program (Diagnostic Instruments Inc., Flint, MI, USA).

### 4.7. Lactoferrin Inhibition by CPE Assay in C7-10 Cells

C7-10 cells dispersed into 12-well plates were grown to 80%–90% confluency, incubated in bLF (Sigma-Aldrich, The Woodlands, TX, USA) for 1 h at RT on a platform rocker in dilutions of; 100, 200, and 300 µg/mL in EMEM supplemented with 10% FBS. Following pretreatment, bLF/EMEM was removed and AR339 or TR339 at equivalent M.O.I. were adsorbed to cells for one h for each bLF concentrations. Cells were washed five times with PBS, EMEM was added, incubated at 28 °C and CPE was monitored at 24 and 48 h p.i.

### 4.8. Lactoferrin Inhibition by Plaque Assay in C7-10 Cells

C7-10 cells in EMEM were plated into 25 cm^2^ tissue culture flasks and grown to 80%–90% confluency as described above. Following initial testing, 200 µg/mL bLF was sufficient and cells were incubated in bLF in EMEM, or EMEM alone for 1 h at RT on a gentle rocker platform. Pre-incubation media was removed and SINVTaV-GFP-TC virus suspended in 2% FBS/PBS with 200 µg/mL bLF, or 2% FBS/PBS alone was added to the cells at a M.O.I. of 0.1. After 1 h at RT, virus-containing medium was removed from the monolayers and cells were washed 3 times with PBS. Cells were overlaid with a 1:1 dilution of 2× EMEM with or without 400 µg/mL bLF in 2% agarose. Flasks were incubated at 28 °C, and monitored every 24 h by fluorescence confocal microscopy for the presence of plaques.

### 4.9. Fluorescent Virus Infection of Mosquitoes and Confocal Microscopy

Adult female, free-mated mosquitoes were fed a virus/blood suspension at 10 days post-emergence via a modified artificial membrane feeder [44]. Mosquitoes were proffered a viremic blood meal with a final titer of 1.5 × 10^7^ pfu/mL SINVTaV-GFP-TC [24] and engorged females were incubated for 30 days at insectary conditions. To harvest SG’s, mosquitoes were placed on a glass petri dish submerged under cold 0.1 M Na cacodylate buffer while viewed through a Leica dissection stereomicroscope. Thumb forceps and insect pins were used to remove the SG’s, which were individually transferred into 96 well plates and incubated in 4% paraformaldehyde/0.1 M Na cacodylate buffer overnight at 4 °C. Salivary glands were surveyed on an Olympus FV1000 confocal microscope (Olympus Corporation, Center Valley, PA, USA) with a 4× objective. Positive-stained glands were moved to welled glass microscope slides in 60% glycerol, coverslips applied and reimaged at higher magnifications.
